# Unusual sulfur isotope effect and extremely high critical temperature in H_3_S superconductor

**DOI:** 10.1038/s41598-018-24442-8

**Published:** 2018-04-16

**Authors:** Radosław Szczęśniak, Artur P. Durajski

**Affiliations:** Institute of Physics, Cz ęstochowa University of Technology, Ave. Armii Krajowej 19, 42-200 Częstochowa, Poland

## Abstract

Recent experiments have set a new record for the transition temperature at which a material (hydrogen sulfide, H_3_S) becomes superconducting. Moreover, a pronounced isotope shift of *T*_*C*_ in D_3_S is evidence of an existence of phonon-mediated pairing mechanism of superconductivity that is consistent with the well established Bardeen-Cooper-Schrieffer scenario. Herein, we reported a theoretical studies of the influence of the substitution of ^32^S atoms by the heavier isotopes ^33^S, ^34^S and ^36^S on the electronic properties, lattice dynamics and superconducting critical temperature of H_3_S. There are two equally fundamental results presented in this paper. The first one is an anomalous sulfur-derived superconducting isotope effect, which, if observed experimentally, will be subsequent argument that proves to the classical electron-phonon interaction. The second one is fact that critical temperature rise to extremely high value of 242 K for H_3_^36^S at 155 GPa. This result brings us closer to the room temperature superconductivity.

## Introduction

**Figure 1:**
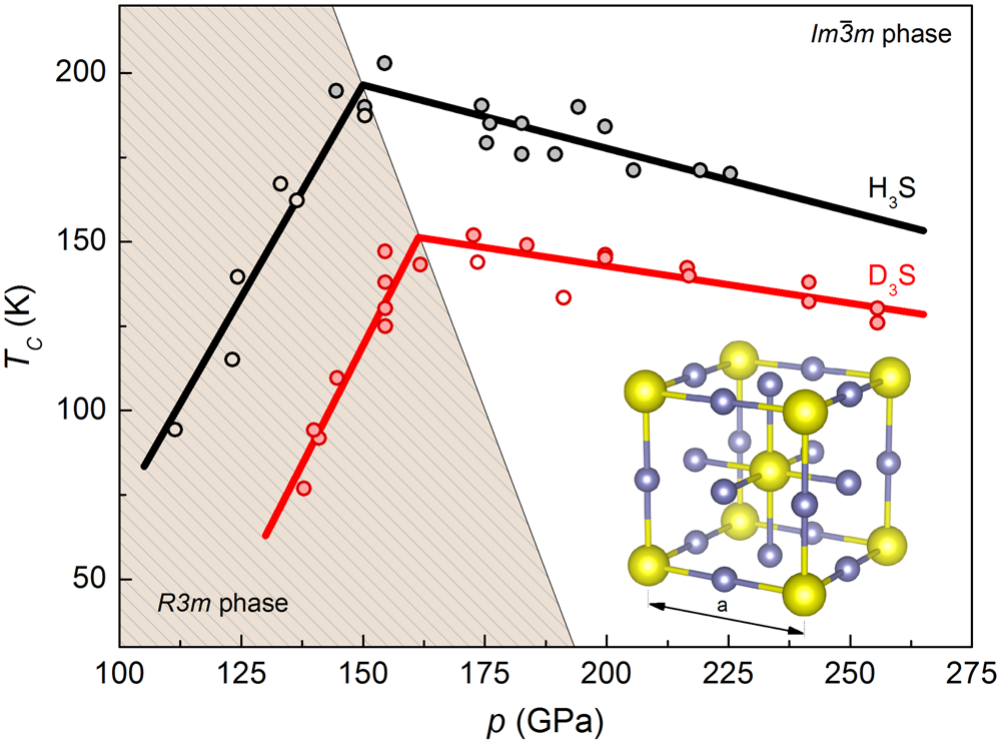
The experimental values of the critical temperature for H_3_S and D_3_S^5,8^. Inset presents the $Im\overline{3}m$ crystal structure for which the record high *T*_*C*_ was observed^8,35^.

According to the Bardeen-Cooper-Schrieffer (BCS) theory of superconductivity^1,2^, achieving high-critical temperature (*T*_*C*_) requires the simultaneous presence of high-frequency phonon modes, large electron-phonon coupling and high electronic density of states (DOS) at the Fermi level^3^. These conditions are fully met in pristine hydrogen and in some previously studied hydrogen-rich systems^4–14^. Back in the year 1968, Ashcroft first proposed that solid hydrogen would be metallized at high pressure and has the potential to be a room-temperature superconductor^15^. Later, due to the fact that pressure of pristine hydrogen metallization was too high for experimental verification (~500 GPa^16^), Ashcroft extended his idea into hydrogen-rich compounds^17^. These materials were expected retain the superconducting properties of pure hydrogen but metallize at pressures significantly lower and reachable by current diamond anvil cell compression technique owing to the *chemical pre-compression*^5,16^. There is an essential hope that these materials can conduct electrical current at room-temperature without loss of energy^18–21^. Based on this idea, a lot of extensive theoretical studies have been carried out^22–26^. Most importantly, this has been confirmed experimentally that compressed hydrogen sulfide (H_3_S) becomes metallic and has a highest superconducting critical temperature ever recorded (203 K at 155 GPa)^5,8^. The experimental relationship between critical temperature and pressure is presented in Fig. 1. Moreover, the directly observed expulsion of external magnetic field from compressed H_3_S superconducting sample (Meissner effect) and the strong isotope shift of *T*_*C*_ in sulfur deuteride (isotope effect) suggest that we deal with a conventional phonon-mediated superconductivity^5,27^. Recently, Bianconi *et al*. suggested that H_3_S is a multiband metal and Lifshitz transitions could occur by increasing pressure^28,29^, however, these assumptions have not been confirmed or disapproved yet in experimental measurements.

Currently, the extensive researches on the possibility of increasing the value of *T*_*C*_ to room temperature are conducting. Recently, generalizing the results on the whole family of the H_*n*_S-type compounds, we shown that the maximum value of *T*_*C*_ can be equal to ~290 K^30^. Unfortunately, neither the pressure increase^31^ nor the taking into account the non-adiabatic effects^32^ do not allow to break the record established by the team led by Eremets^5^. Continuing the earlier study, in this paper we take into account the previously completely ignored pathway to increase transition temperature and set a new record for superconductivity. We investigate the influence of the sulfur isotope effect on the superconducting state in compressed hydrogen sulfide.

## Theoretical model and computational methods

In this paper, the electronic properties, lattice dynamics and electron-phonon coupling were calculated using the first-principle pseudopotential plane-wave method based on the density functional theory through the Quantum-Espresso package^33,34^. The ultrasoft Vanderbilt pseudopotentials for S and H atoms were employed with a kinetic energy cut-off equal to 80 Ry. The electronic band structure calculations were performed for 32 × 32 × 32 Monkhorst-Pack *k*-mesh to sample the Brillouin zone with the Gaussian smearing of 0.03 Ry. The phonon dispersion and electron-phonon coupling matrices were computed within the framework of the linear-response method on the set of 8 × 8 × 8 **q**-point mesh for the first Brillouin zone integrations. The energy convergence and precision of all presented results are controlled by assuming the sufficiently small (10^−16^ Ry) threshold on the change in total energy.

**Figure 2:**
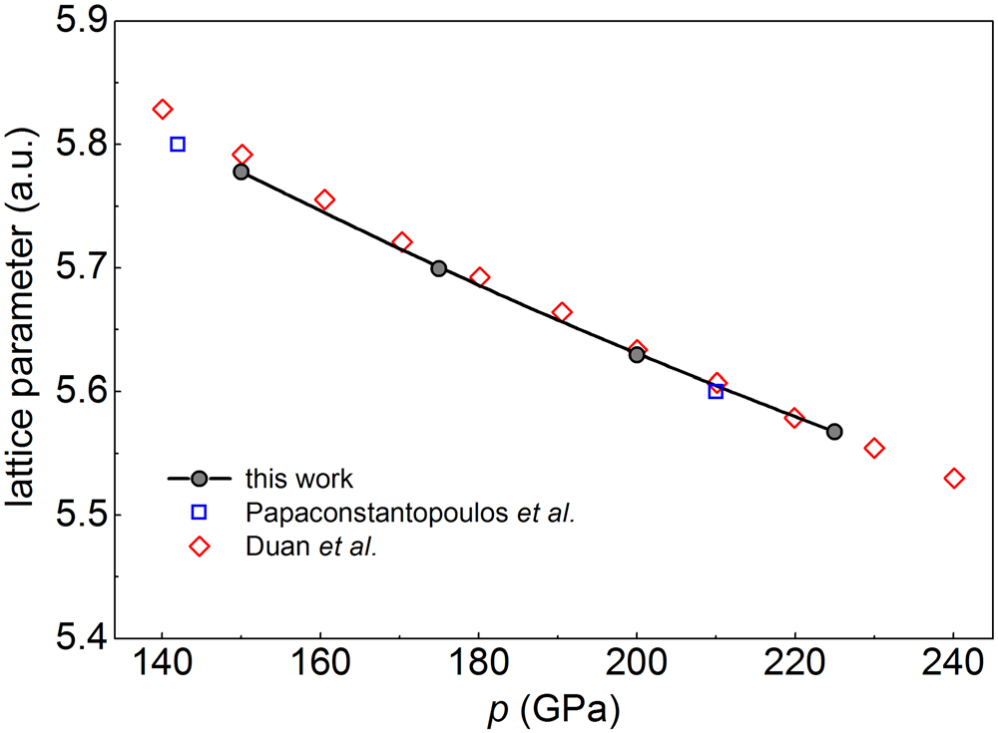
Calculated lattice constant as a function of pressure. The open symbols correspond to the previously reported theoretical results^35,36^.

The X-ray diffraction experiments^8^ confirm that the superconducting phase of H_3_S is in good agreement with the theoretically predicted body-centred cubic $Im\overline{3}m$ structure^35^. The ball-and-stick model of cubic H_3_S structure under compression is shown in the inset of Fig. 1. To evaluate the lattice constant and phase stability of the investigated $Im\overline{3}m$ structure we performed total energy calculations and structural relaxations in a wide range of high pressure. The lattice constant and atomic positions were relaxed according to the atomic forces. This procedure was repeated until the forces on every atom of the unit cell were less than 1 meV/a.u. and the resulting stress less than 1 kbar. In this way, the fully relaxed structural parameters of H_3_S have been obtained. In Fig. 2, the calculated lattice constant as a function of pressure was presented and compared with other theoretical predictions^35,36^. These results coincide with very good accuracy with previous reports, consequently, for the pressure range where the high-*T*_*C*_ was measured (155–225 GPa) we assume that $Im\overline{3}m$ structure is stable and we expect that the change of sulfur isotope mass can significantly influences on the superconducting state of H_3_S.

## Results and Discussion

**Figure 3:**
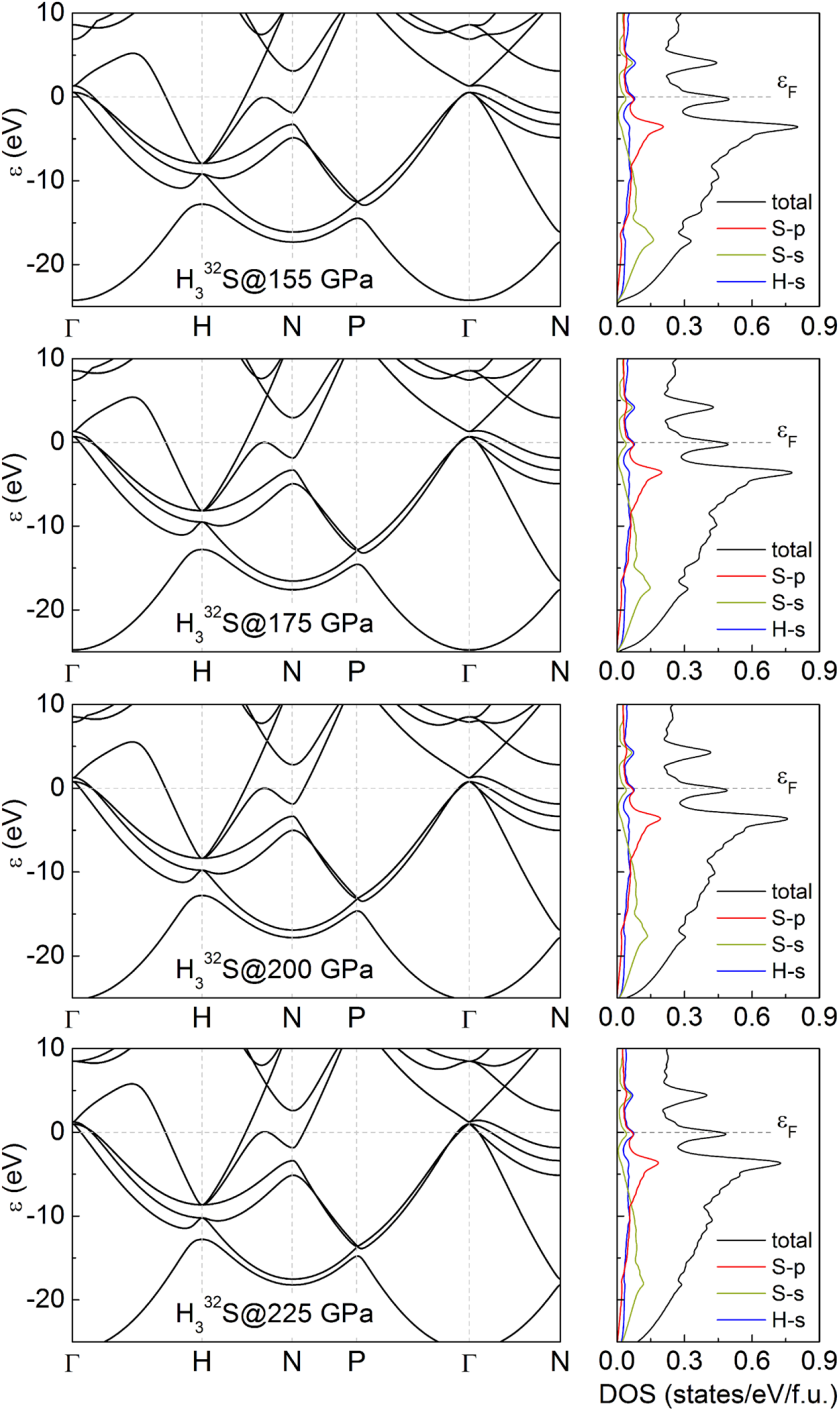
Calculated electronic band structure and partial density of states (DOS) for H_3_^32^S at selected pressures.

To analyze the electronic properties of H_3_S the electronic band structure and the partial density of states (DOS) were calculated. In Fig. 3 we can see the results for investigated pressures 155, 175, 200 and 225 GPa and for one of stable sulfur isotope ^32^S (94.99% natural abundance). The existence of electrons in the Fermi level indicates the metallic character of all cases. The van Hove singularity near the Fermi level can enhance the electron-phonon coupling strength and hence can be responsible for high-temperature superconductivity. Furthermore, very similar shape of electronic band structure and DOS are found in whole range of pressure. Also the change of sulfur isotope in elemental cell has no effect on the electronic properties of studied system. On this basis, we can suppose that phonons properties in hydrogen sulfide systems are actually responsible for change in their thermodynamic properties.

**Figure 4:**
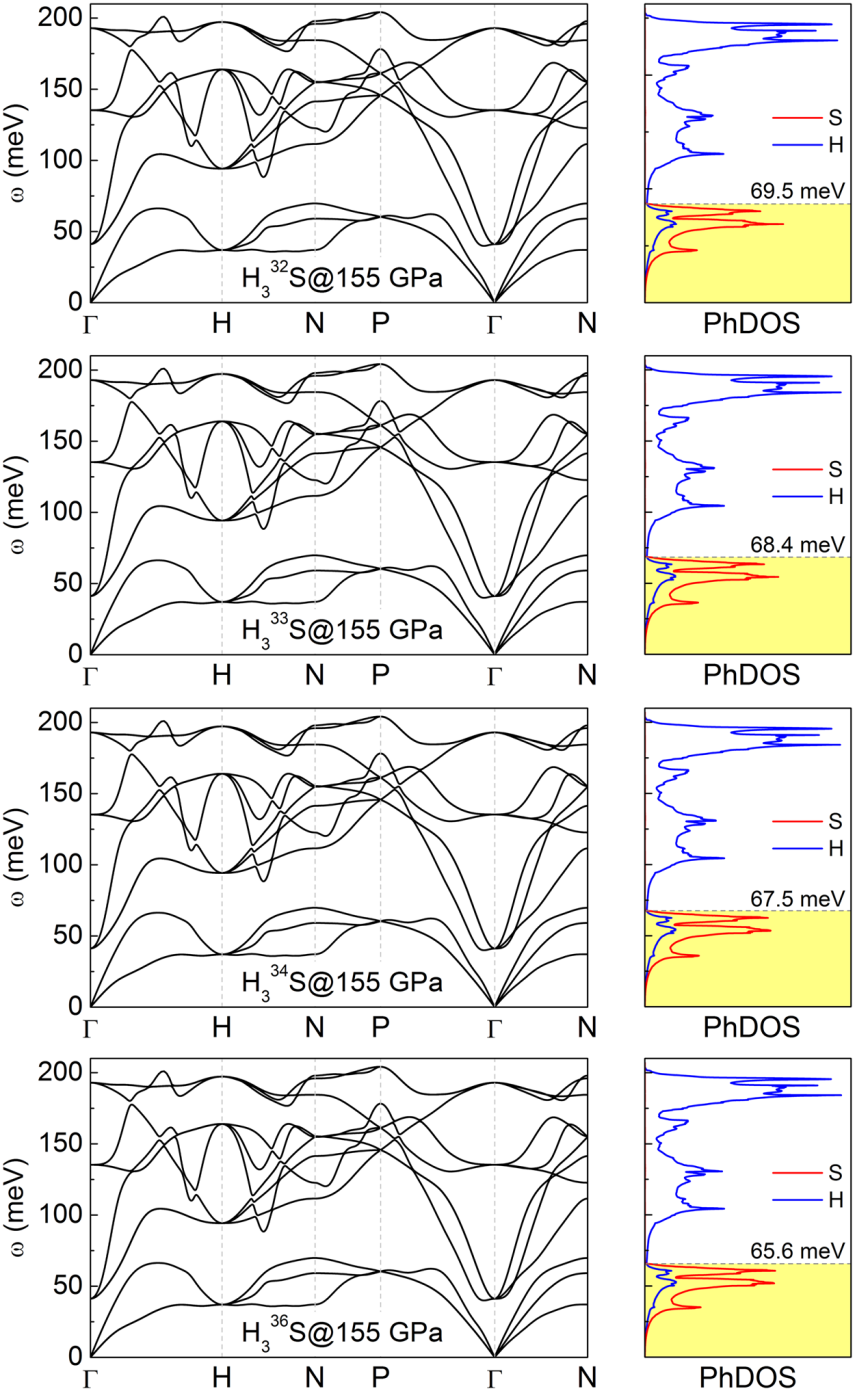
Phonon dispersion and projected phonon density of states (PhDOS) for H_3_S with all stable sulfur isotopes. Results for pressure of 155 GPa.

Figure 4 shows the calculated phonon band structure and projected phonon density of states (PhDOS). Phonon calculations did not give any imaginary frequency vibration mode in the whole Brillouin zone, indicating the dynamic stability of $Im\overline{3}m$ structure. Based on the PhDOS, we found that the vibration frequency is divided into two parts as a result of the different atomic masses of S and H atoms. The low-frequency bands mainly result from the vibrations of the S atoms, whereas the H atoms are mostly related to vibrations with higher frequency modes. Note that the contribution derived from sulfur is shifted towards the lower frequencies together with increasing S isotope mass. This should be reflected in the shape of the Eliashberg electron-phonon spectral function *α*^2^*F*(*ω*), which weights the phonon density of states with the coupling strengths and appropriately describes the pairing interaction due to phonons:

1 $${\alpha }^{2}(\omega )F(\omega )=\frac{1}{2\pi N(0)}\sum _{{\bf{q}}{\rm{\nu }}}\delta (\omega -{\omega }_{{\bf{q}}{\rm{\nu }}})\frac{{\gamma }_{{\bf{q}}{\rm{\nu }}}}{\hslash {\omega }_{{\bf{q}}{\rm{\nu }}}},$$

where

2 $$\begin{array}{l}{\gamma }_{{\bf{q}}\nu }=\pi {\omega }_{{\bf{q}}\nu }\sum _{ij}\int \frac{{{\rm{d}}}^{{\rm{3}}}k}{{{\rm{\Omega }}}_{BZ}}|{g}_{{\bf{q}}\nu }({\bf{k}},i,j{)|}^{2}\delta ({\varepsilon }_{{\bf{q}},i}-{\varepsilon }_{F})\delta ({\varepsilon }_{{\bf{k}}+{\bf{q}},j}-{\varepsilon }_{F}\mathrm{).}\end{array}$$

Symbols *N*(0), *γ*_**q**ν_, and *g*_**q**ν_(**k**, *i*, *j*) denote the density of states at the Fermi energy, the phonon linewidth, and electron-phonon matrix elements, respectively.

**Figure 5:**
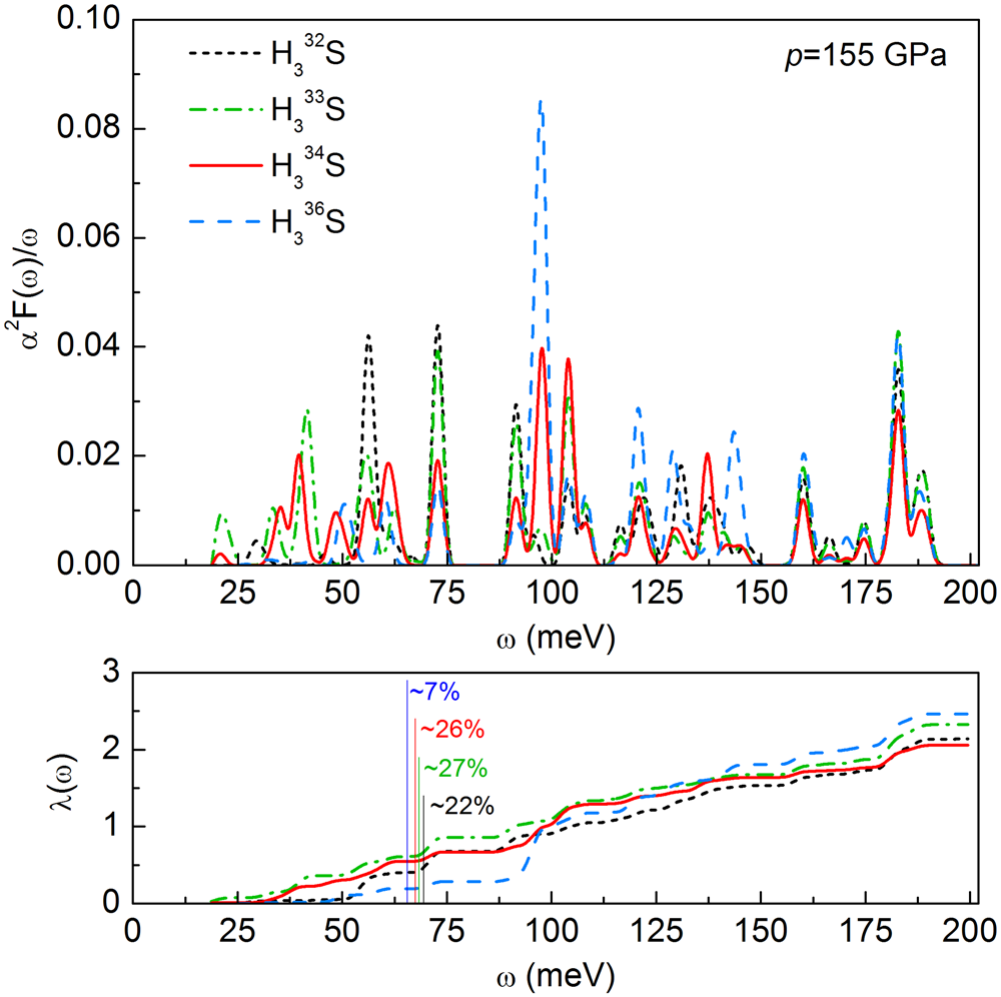
The Eliashberg spectral functions for H_3_S at 155 GPa. Only the stable S isotopes was investigated (upper panel). The electron-phonon coupling parameter integral as a function of frequency (bottom panel). The percent contribution of *λ* originating from sulfur was marked for all cases.

The Eliashberg spectral function, electron-phonon coupling constant *λ* and logarithmic average phonon frequency *ω*_l*n*_ are investigated to explore the possible record critical temperature of H_3_S. The calculated *α*^2^*F*(*ω*)/*ω* functions and integration of *λ* for H_3_^32^S, H_3_^33^S, H_3_^34^S and H_3_^36^S at 155 GPa are shown in Fig. 5. The main contribution to the electron-phonon coupling constant derived from hydrogen and it should be highlights that the H atoms play a significant role in the superconductivity of hydrogen sulfide. For H_3_^32^S nearly 22% of *λ* originates from sulfur. With increasing mass of sulfur isotope, it is very interesting to note that, the part coming from S changes and finally decreases to 7% for H_3_^36^S. The comparison of Eliashberg functions with phonon density of states shows that the square of the matrix element of the electron-phonon interaction averaged over the Fermi surface *α*^2^(*ω*) is responsible for complicated shape of the Eliashberg spectral functions. This may leads directly to the non-monotonic changes of magnitudes related to *α*^2^*F*(*ω*) such as *λ*, *ω*_*ln*_, and critical temperature with increasing mass of sulfur isotope.

The high vibrational phonon frequency and the strong electron-phonon coupling constant lead directly to a high superconducting critical temperature which was calculated using the Eliashberg formalism^37,38^. It should be noted that in literature *T*_*C*_ is usually obtained using the simple approach proposed by McMillan or Allen and Dynes^39,40^, which represent the weak-coupling limit of the more elaborate Eliashberg approach^37^. In our previous papers^41,42^, we proved that the McMillan or Allen-Dynes-modified McMillan formulas and Eliashberg equations lead to similar results for small *λ* and Coulomb pseudopotential *μ*^★^. For larger *λ* and *μ*^★^, however, the analytical formulas predicts underestimated *T*_*C*_ values. In the case of the hydrogen sulfide the electron-phonon interaction is strong, hence the analytical formulas are inappropriate. The isotropic Migdal-Eliashberg equations were solved in a numerical way^43^ using 2201 Matsubara frequencies *ω*_*n*_ = (*π*/*β*)(2*n*−1), where *n* = 0, ±1, ±2, …, ±1100, and a Coulomb pseudopotential which was chosen to match the measured value of *T*_*C*_ for standard S atomic weight of 32.06 u^44^.

**Figure 6:**
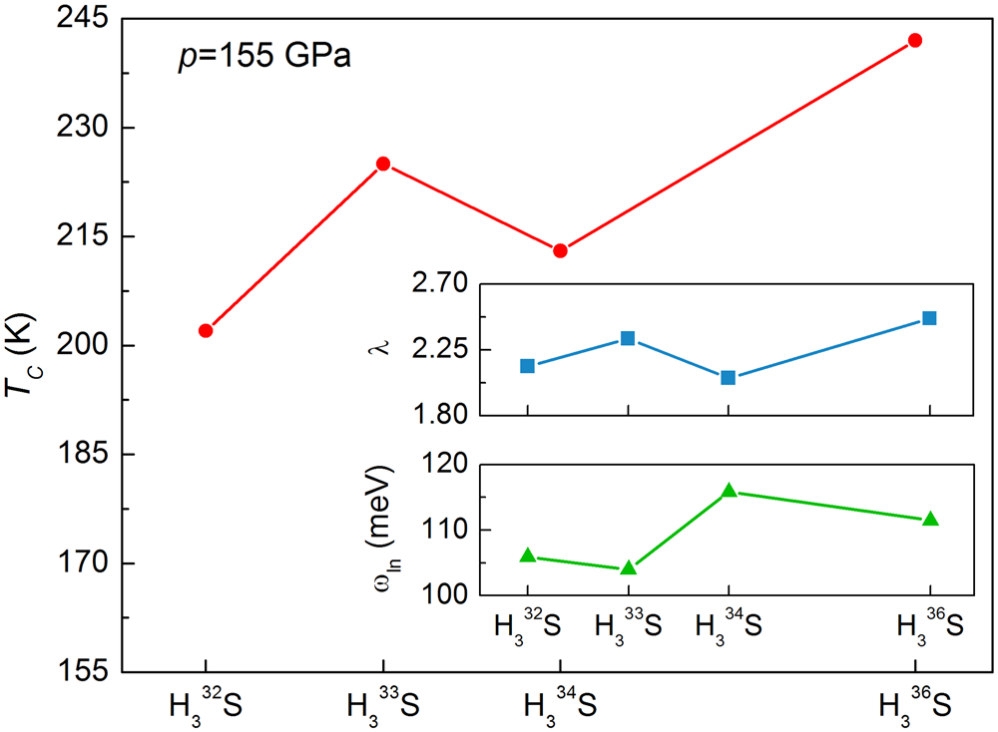
Critical temperature calculated for investigated systems at 155 GPa. Insets present behavior of *λ* and *ω*_l*n*_.

Such an assumption ensures the stability of the numerical solutions for *T* ≥ 1 K. The superconducting transition temperature was estimated to be in the range of 202–242 K at 155 GPa. The calculated *T*_*C*_, *λ* and *ω*_l*n*_ for H_3_^32^S, H_3_^33^S, H_3_^34^S and H_3_^36^S at 155 GPa are summarized in Fig. 6. It is very interesting to note that *T*_*C*_ is strongly correlated with *λ* and despite decrease in *ω*_l*n*_ for H_3_^36^S, *λ* increases resulting in an enhanced *T*_*C*_ to record value of 242 K.

**Figure 7:**
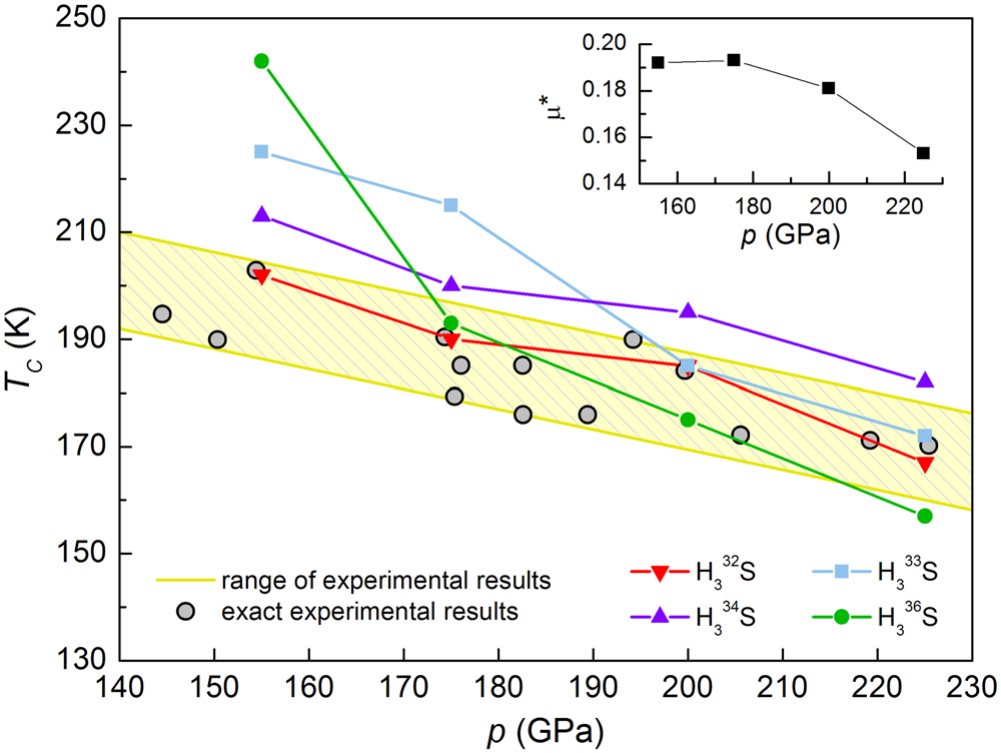
Pressure dependence of the superconducting critical temperature measured^5,8^ and calculated for stable isotopes of sulfur. Inset presents the Coulomb pseudopotential reproducing the experimental value of *T*_*C*_ for standard S atomic weight of 32.06 u.

The isotope effect of superconducting critical temperature is best described in terms of the isotope effect coefficient *α*. For experimental results of hydrogen and deuterium sulfide at *p* = 155 GPa we have *α* = 0.47^23^. This value is very close to the theoretical value of 0.5 predicted within the framework of the BCS scenario. In this paper, for the most extreme case of sulfur isotopes at 155 GPa we have the following relation:

3 $$\alpha =-\frac{{\rm{l}}{\rm{n}}\,{[{T}_{C}]}_{{{{\rm{H}}}_{3}}^{36}{\rm{S}}}-\,{\rm{l}}{\rm{n}}\,{[{T}_{C}]}_{{{{\rm{H}}}_{3}}^{32}{\rm{S}}}}{{\rm{l}}{\rm{n}}\,{[M]}_{{}^{36}{\rm{S}}}-\,{\rm{l}}{\rm{n}}\,{[M]}_{{}^{32}{\rm{S}}}},$$

where ${[M]}_{{}^{32}{\rm{S}}}$ and ${[M]}_{{}^{36}{\rm{S}}}$ are the atomic mass of ^32^S and ^32^S isotope, respectively. Contrary to most superconducting materials, the calculated isotope coefficient is negative *α* = −1.5. The inverse and nontrivial behavior can be also observed for other isotopes and higher pressures, as shown in Fig. 7. On the other hand, some other systems also display values that are smaller than zero. For example the inverse superconducting isotope coefficient has been observed in uranium (*α* = −2)^45^, metal hydride PdH (*α* = −0.25)^46^ or lithium where *α* sign changes with increasing pressure^47^. Let us strongly emphasize, however, that the isotope effect in superconductivity is taken as evidence for phonon mediation. Coming back to the Fig. 7, we can additionally observed that with increasing pressure the critical temperature decreasing which is in a general agreement with the trend established by the experimental results. Moreover, it should be emphasized that correctness of our methods and numerical calculations was confirmed by comparison the obtained results with the previous ones for the natural isotope concentration of sulfur^12,13,35,48^. To benchmark the validity of the results obtained in the present work, in next section we have shown the calculated electronic structure and phonon dispersions together with the results previously reported by Duan *et al*.^35^. Moreover, we examined the isotope effect for H_3_S and D_3_S.

## Proving correctness of the presented results

**Figure 8:**
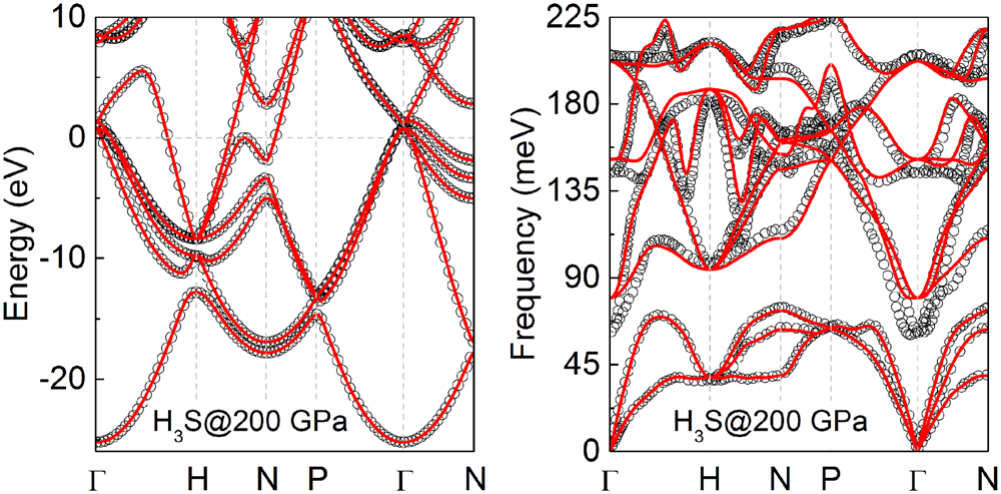
Comparison between our results (red lines) obtained for the natural isotope concentration of sulfur and the results previously reported by Duan *et al*.^35^ (open circles).

A benchmark study on correctness of our results was done to electronic structure and phonon dispersions which were collate with the results previously reported by Duan *et al*.^35^. This comparison for the natural isotope concentration of sulfur in H_3_S at 200 GPa was shown in Fig. 8. On this base we can found that here is almost exact coincidence which proves the correctness of the results reported in the present work.

**Figure 9:**
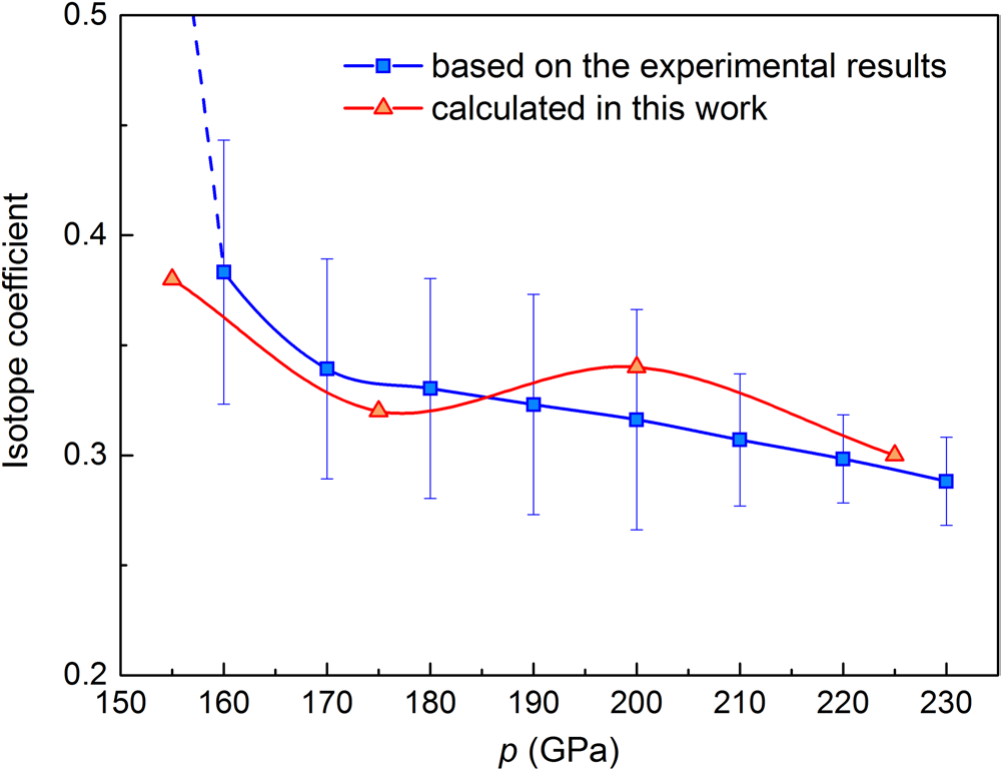
The isotope coefficient as a function of pressure calculated from the averaged experimental critical temperature of H_3_S and D_3_S at the same pressure (blue square) and our results (red triangles) obtained using Eliashberg formalism with Coulomb pseudopotential estimated for standard *S* atomic weight.

Moreover, we compared the isotope coefficient resulting from the experimental critical temperature of H_3_S and D_3_S (see Fig. 1) with our estimations conducted within the framework of the Eliashberg formalism. In both cases the isotope coefficient decreases with pressure which is connected with decreasing difference between critical temperature for H_3_S and D_3_S. As shown in Fig. 9, the very high level of consistency was achieved. This is another argument in favor of correctness and high-value of presented herein results.

## Conclusion

We reported the influence of the substitution of ^32^S atoms by the heavier isotopes ^33^S, ^34^S and ^36^S on the electronic properties, lattice dynamics and superconducting critical temperature in H_3_S. We observe that for a pressure of 155 GPa this substitution causes a strong (20%) enhancement of *T*_*C*_ from 202 to 242 K. This unexpectedly high *T*_*C*_ far exceeds the previous record of 203 K and bring us closer to achieving room-temperature superconductivity in hydrogen-rich materials at high pressure. The second very important and interesting result, reported in this paper, is uncommon sulfur isotope effect. We noted the strong negative isotope coefficient (*α* =−1.5) between H_3_^32^S and H_3_^36^S, and variation of the isotope effect with the increasing pressure.

We expected that our significant findings can stimulate future high-pressure experiments and that suggested pathway to increase *T*_*C*_ can be appropriate to reach near-room-temperature superconductivity.
